# Differentially Expressed Genes in Hepatopancreas of Acute Hepatopancreatic Necrosis Disease Tolerant and Susceptible Shrimp (*Penaeus vannamei)*

**DOI:** 10.3389/fimmu.2021.634152

**Published:** 2021-05-13

**Authors:** Hung N Mai, Luis Fernando Aranguren Caro, Roberto Cruz-Flores, Brenda Noble White, Arun K. Dhar

**Affiliations:** Aquaculture Pathology Laboratory, School of Animal & Comparative Biomedical Sciences, The University of Arizona, Tucson, AZ, United States

**Keywords:** AHPND, *Penaeus vannamei*, AHPND tolerant P vannamei, shrimp immunity, immune genes

## Abstract

Acute hepatopancreatic necrosis disease (AHPND) is a lethal disease in marine shrimp that has caused large-scale mortalities in shrimp aquaculture in Asia and the Americas. The etiologic agent is a pathogenic *Vibrio* sp. carrying binary toxin genes, *pir*A and *pir*B in plasmid DNA. Developing AHPND tolerant shrimp lines is one of the prophylactic approaches to combat this disease. A selected genetic line of *Penaeus vannamei* was found to be tolerant to AHPND during screening for disease resistance. The mRNA expression of twelve immune and metabolic genes known to be involved in bacterial pathogenesis were measured by quantitative RT-PCR in two populations of shrimp, namely P1 that showed susceptibility to AHPND, and P2 that showed tolerance to AHPND. Among these genes, the mRNA expression of chymotrypsin A (ChyA) and serine protease (SP), genes that are involved in metabolism, and crustin-P (CRSTP) and prophenol oxidase activation system 2 (PPAE2), genes involved in bacterial pathogenesis in shrimp, showed differential expression between the two populations. The differential expression of these genes shed light on the mechanism of tolerance against AHPND and these genes can potentially serve as candidate markers for tolerance/susceptibility to AHPND in *P. vannamei*. This is the first report of a comparison of the mRNA expression profiles of AHPND tolerant and susceptible lines of *P. vannamei*.

## Introduction

Acute hepatopancreatic necrosis disease (AHPND) is a lethal disease of marine shrimp that emerged in China in 2009. Since then it was reported in other countries including Vietnam, Thailand, Mexico, Philippines, Bangladesh, US and South-Korea (1–7). The etiologic agent was initially identified as *Vibrio parahaemolyticus* carrying plasmid-borne binary toxin genes, *pir*A and *pir*B (8, 9). Subsequently other species of *Vibrio* including *V. harveyi* and *V. owensii* have been reported to cause AHPND (10, 11). The clinical signs of AHPND include atrophy and pale discoloration of hepatopancreas, soft shell, gut with discontinuous or no content, and often 100% of mortality occurs in shrimp farms experiencing AHPND outbreaks (12–14). Histopathology of the hepatopancreas tissue from AHPND affected shrimp reveals three different stages of disease development, namely initial, acute/terminal and chronic phases. In the initial phase, elongation of epithelial cells in hepatopancreas is common, whereas during the acute/terminal phase necrosis of tubular epithelial cells and inflammatory responses are observed (13). The chronic phase of AHPND is characterized by the epithelial necrosis and bacterial inflammation in the tubules which is similar to septic hepatopancreatic necrosis (SHPN) (15).

It has widely been accepted that shrimp protect themselves from microbial pathogens by innate immunity that encompass humoral and cellular responses. Recently, it has been reported that the PirA*^VP^*- and PirB*^VP^* binary toxin encoded by *V. parahaemolyticus* can be neutralized by either hemocyanin or anti-lipopolysaccharide factor (16). In addition, the interaction between immune and metabolism appears to play a role in AHPND response in hepatopancrease in shrimp (17, 18). Until now, due to the lack of AHPND- tolerant lines of shrimp, no effort could be made to examine the gene expression profiles of AHPND- susceptible and tolerant lines of shrimp.

Recently, a line of *P. vannamei* shrimp has been identified that displays tolerance to AHPND (15). In this study, we initially compared the mRNA expression of twelve metabolic/immune related genes between AHPND-tolerant and-susceptible lines of *P. vannamei* by reverse transcriptase quantitative PCR (RT-qPCR). These genes are known to be involved in bacterial pathogenesis (19–24). The expression of seven genes that showed statistically significant differences between the tolerant and susceptible lines were validated. The results showed that there is a significant difference in the expression of these genes between AHPND-tolerant and-susceptible lines of *P. vannamei*. The potential implications of differential expression of these genes are discussed in the context of immunity and pathogenesis.

## Materials and Methods

### Bioassay 1- Assessing AHPND Tolerance in *P. vannamei*

*Vibrio parahemolyticus* (Strain 13-028A/3) (Vp_AHPND_) was used for the experimental challenges following a previously published protocol (15). Three *P. vannamei* family lines were obtained from a commercial supplier as a part of an on-going family line screening for AHPND-tolerance. Each line was stocked separately into a total of nine 1000 L tanks in triplicate. From each of these three lines, we screened 56, 57 and 78 organisms from lines one, two and three respectively. A Specific Pathogen Free (SPF) *P. vannamei* (N=60, average weight 3 g) (AHPND susceptible line, population P1, were obtained from a commercial supplier in the USA and stocked into two 90L tanks and used as positive controls for AHPND challenge. A third tank containing SPF shrimps of the same genetic line (N=64) was used as negative control. An immersion challenge was performed using an inoculum load of 10^6^ cfu/ml (15). The experiment was terminated at 7 days post-inoculation. The mortality in each tank was recorded daily, and a subset of moribund and surviving animals was examined by routine H&E histology.

### Histopathology

Moribund *P. vannamei* were fixed in Davidson’s alcohol-formalin-acetic acid (AFA) fixative. The samples were processed, embedded in paraffin, sectioned (4 μm thick) and analyzed in accordance with standard methods (25).

### Bioassay 2- Initial Gene Expression Analyses

To evaluate gene expression in the AHPND-tolerant and-susceptible lines, a second challenge was conducted. A total of 46 animals were utilized from P1 (n= 42, average weight 5g) with 21 animals challenged with VP_AHPND_, 21 animals utilized as negative controls and 4 animals sampled for gene expression analysis prior to challenge. The tolerant line was named population P2 (n=20, average weight 9g), with 9 animals challenged with VP_AHPND_, 9 unchallenged as negative controls and 2 animals sampled for gene expression analysis prior to challenge. All shrimp from each family were tagged in the 4th abdominal segment with uniquely colored elastomer tags to allow visual identification. Both families were then held in a single 1000L tank and challenged by immersion, as described above. The negative controls were held in a single 1000L tank but were not challenged.

Twenty-four hours after challenge, animals were collected for gene expression analysis. Limited numbers (N=4) of the susceptible P1 line were available for sampling due to the typically high mortality rates observed in AHPND challenges in the first 24 hours. Two shrimp were collected from the tolerant population P2 at 24 hours post-infection to leave enough animals in the tank for a survival comparison at termination. In order to keep the sample numbers equal, a pool of 10 challenged animals from P2 group in the initial family line challenge were pooled as 2 samples for a total of 4 samples.

### Measuring the Expression Levels of Candidate Immune and Metabolic Genes in AHPND Challenged *P. vannamei*

Four shrimp samples per population were collected from each treatment for RNA extraction using RNAzol following the manufacturer’s protocol (MRC, Ohio, USA). The RNA was treated with DNase 1 (Invitrogen, USA). One µg of DNase treated RNA was used for cDNA synthesis using Superscript IV and following the manufacturer’s recommendation (Invitrogen, USA). After that, cDNA was subjected to gene expression analysis.

The mRNA expression of β-glucan binding protein (BGBP) (AY249858.1), Crustin-P (CRSTP) (AY488497), C-type lectin 1-like (CTL1-like) (DQ858900.1), Extracellular Copper/Zinc Superoxide dismutase (EC-SOD) (HM371157), Kazal protease inhibitor (KPI) (AY544986), lipopolysaccharide and β-1,3-glucan-binding protein (LGBP) (EU102286.1), Penaidin 2 (PEN2) (Y14925), Prophenol oxidase activation system 2 (PPAE2) (AWF98992.1), Serpin8 (SEP8) (KU853046.1), serine protease (SP) (AY368151), Chymotrypsin A (ChyA) (Y10664), Chymotrypsin B (ChyB) (Y10665) and two toxin genes of *V. parahaemolyticus* (i.e. *pir*A and *pir*B toxin genes, KM067908) were measured by quantitative PCR (StepOnePlus Real-time PCR system, Applied biosystems, USA) using PowerUPTM SYBR Green Master Mix (Applied Biosystem, USA). The reaction mixture contained 10 µl of PowerUP™ SYBR Green Master Mix, 0.8 µl (0.4 µM) of each primer, 2.0 µl of cDNA and 6.4 µl of sterile water in a reaction volume of 20 µl. The thermal profile for the reaction was 2 minutes at 50°C, 2 minutes at 95°C followed by 40 cycles of 3 seconds at 95°C and 30 seconds at 60°C. The primer sequence for each gene is given in **Supplementary Table S1**. In addition, literature search of the responses to pathogens of studied genes was provided in **Supplementary Table S4**. Each sample was run in duplicate and the mean Ct value was used for gene expression analysis. Expression levels of each gene in various populations are shown relative to the expression in corresponding control treatments according to the formula of Livak and Schmittgen (26). For evaluating the gene expression between challenged animals coming from P1 and P2 population, the expression levels of each gene were shown relative to the expression in the P1 negative control. The expression value was converted to Log based 2 prior to statistical analysis. The genes showing statistical difference in expression level in Bioassay 2 were validated in Bioassay 3.

### Bioassay 3- Gene Expression Validation

Two hundred shrimp (average wt. 2 g) with the same genetic characteristics of the AHPND tolerant shrimp line (P2) from Bioassay 2 were stocked in two 1000 L-tanks (100 shrimp/tank) in which one tank was challenged with VP_AHPND_. The remaining tank was used as negative control. Forty SPF shrimp (AHPND susceptible shrimp) (average wt. ~1.5 g) (P1) were stocked in two 90L-tanks (20 shrimp/tank), one tank was challenged with VP_AHPND_. The remaining tank was used as negative control. The AHPND challenge protocol was the same as described previously. After 24 hours, nine shrimp from each tank were collected for gene expression analyses.

### Statistical Analysis

The statistical significances in the difference in Log based 2 value of gene expression between control and challenged animals in each population and between challenged animals in P1 and P2 populations was determined by using Student’s t-test with P<0.05, SPSS v.16 software. The mortality data was analyzed using Kaplan-Meier method, SPSS v.16 software.

## Results

### Bioassay 1- Assessing AHPND Tolerance in *P. vannamei*

**Figure 1:**
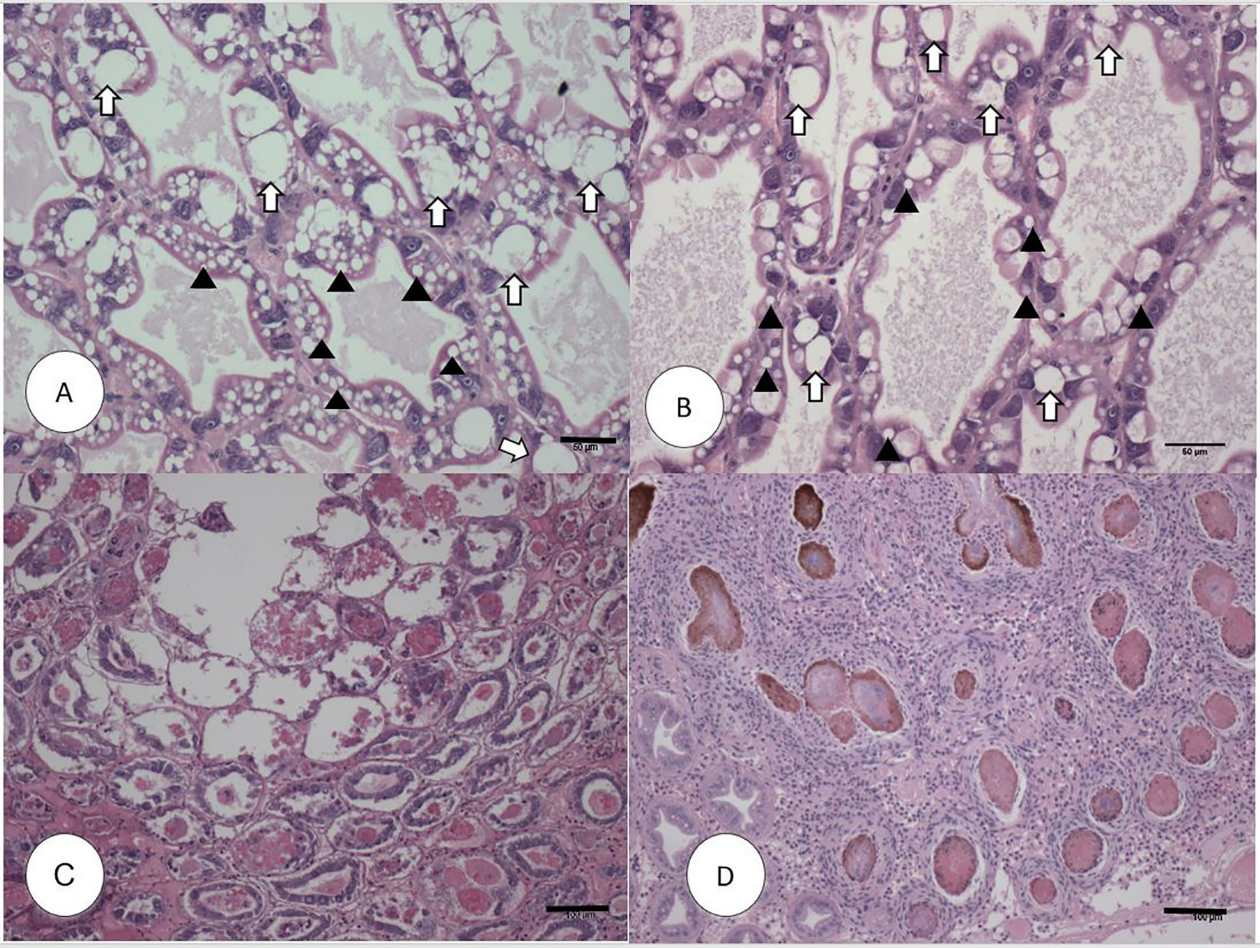
H&E (Mayer–Bennet hematoxylin and eosin-phloxine) histology of *Penaeus vannamei* from Bioassay 1. *Penaeus vannamei* from negative control tank from the P1 **(A)** and P2 populations **(B)** showing intact tubules and epithelial R-cells (arrowhead) and B-cells (white arrow). Acute phase infection of AHPND in shrimp from P1 population **(C)** showing a severe sloughing of epithelial tubule cells into the lumen. Terminal phase of AHPND infection in shrimp from P2 population **(D)** showing a severe intertubular hemocytic infiltration surrounding the affected melanized tubules. Scale bars for **(A**, **B)** = 50 μm; **(C**, **D)** = 100 μm.

In experimental AHPND challenge, the survival rate in the AHPND-tolerant P2 population was over five times higher compared to the AHPND-susceptible P1 population (72% *vs*. 12.5%). Meanwhile, the survival rate in negative control was 95.45% (**Supplementary Figure S1**). The histopathology of the P1 and P2 populations are presented in **Figure 1**. The Davidson-fixed shrimp from the healthy, negative controls from P1 and P2 displayed normal structure of tubules and epithelial cells in the hepatopancreas including high levels of lipid droplets (R-cells), secretory vacuoles (B-cells) and absence of AHPND (**Figures 1A, B**). In contrast, shrimp from the P1 population challenged with Vp_AHPND_ displayed lesions typical of AHPND in the acute phase, including a multifocal necrosis and massive sloughing of HP tubule epithelial cells in the hepatopancreas. At this stage, bacterial colonization was not observed (**Figure 1C**). Shrimp from the P2 population displayed the typical VP_AHPND_ chronic phase characterized by SHPN-like lesions (**Figure 1D**).

### Bioassay 2- Initial Gene Expression Analyses, Measuring the mRNA Expression of Immune and Metabolic Genes in *P. vannamei*

**Figure 2:**
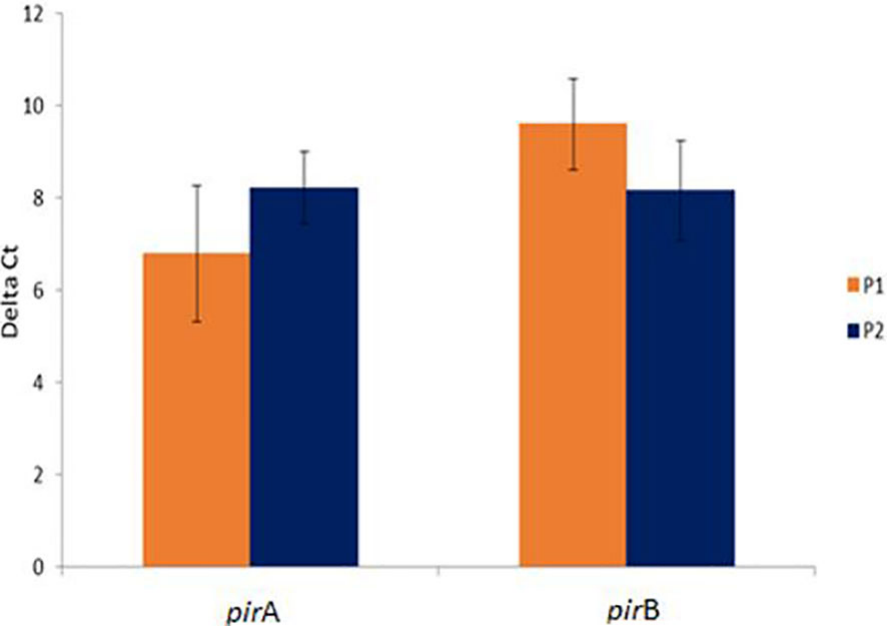
Comparative *pir*A- and *pir*B- toxin genes expression in challenged animals from Bioassay 2 in the P1 and P2 populations. The levels of mRNA expression is expressed as a normalized mean Ct value. The data is presented as ΔMean Ct ± SD. Statistical significance between P1 and P2 populations for each of the candidate gene was determined using Student’s t-test.

Interestingly, there was no significant difference in the expression levels of *pir*A/*pir*B genes between P1 and P2 population (P>0.05) (**Figure 2**).

The mRNA expression of a set of immune and metabolic genes (i.e. SEP8, BGBP, CRSTP, CTL1-like, KPI, LGBP, EC-SOD, PEN2, PPAE2, SP, ChyA and ChyB) were measured by RT-qPCR in control animals and challenged animals in each population. We also compared the levels of gene expression in AHPND susceptible P1 and AHPND tolerant P2 populations.

**Figure 3:**
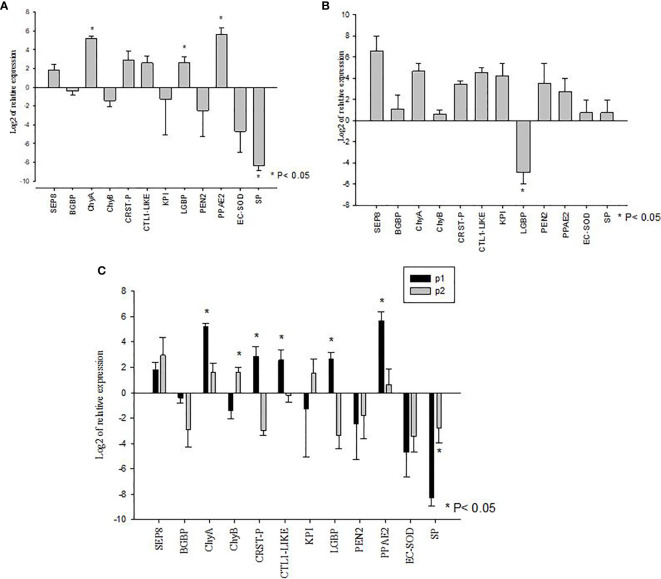
Gene expression profiles of metabolic and immune genes in shrimp *Penaeus vannamei* from Bioassay 2. **(A)** The mRNA expression profile in AHPND susceptible (P1) population following experimental challenge. **(B)** The mRNA expression profile in AHPND tolerant (P2) population following experimental challenge. Expression levels of each gene are shown relative to the expression in correspond control treatment **(C)** Comparison of gene expression profile from AHPND susceptible (P1) vs. AHPND resistant/tolerant (P2) population. Expression levels of each gene are shown relative to the expression in P1 negative control treatment. The data is presented as log2 of relative expression ± SD. Statistical significance between control and challenged animals for each of the candidate gene was determined using Student’s t-test. *P<0.05. BGBP, β-glucan binding protein; CRST P, Crustin P; CTL, C-type lectin 1-like; ECSOD, Extracellular Copper/Zinc Superoxide dismutase; KPI, Kazal protease inhibitor; LGBP, Lipopolysaccharide and β-1,3-glucan-binding protein; PEN2, Penaidin 2; PPAE2, Prophenol oxidase activation system 2; SEP, Serpin8; SP, Serine protease; ChyA, Chymotrypsin A; ChyB, Chymotrypsin B.

The *V. parahaemolyticus* infection led to the significant upregulation of expression of LGBP, PPAE2, and ChyA transcripts in the P1 population (P<0.05) whereas SP mRNA showed significant down regulated expression (P<0.05). The mRNA levels of BGBP, CRSTP, CTL1-like, KPI, PEN2, EC-SOD, SEP8 and ChyB in the challenged and un-challenged groups did not show significant differences (P>0.05) (**Figure 3A**).

In the P2 population, *V. parahaemolyticus* infection led to the significant downregulated expression of LGBP mRNA (P<0.05). The expression of BGBP, SEP8, CTL1-like, CRSTP, KPI, EC-SOD, PPAE2, PEN2, SP, ChyA and ChyB showed no significant difference between challenged and un-challenged groups (P>0.05) (**Figure 3B**).

When the mRNA expression levels were compared between AHPND challenged animals from the P1 and P2 populations, there were significant differences in the expression profiles of some genes. For example, while the susceptible P1 population showed significantly higher levels of expression of ChyA, CRSTP, CTL1-like, LGBP and PPAE2 (P<0.05) (**Figure 3C**), the AHPND-tolerant population P2 showed higher expression of SP and ChyB compared to the susceptible P1 population (P<0.05) (**Figure 3C**) (**Supplementary Table S2**).

### Bioassay 3- Gene Expression Validation

**Figure 4:**
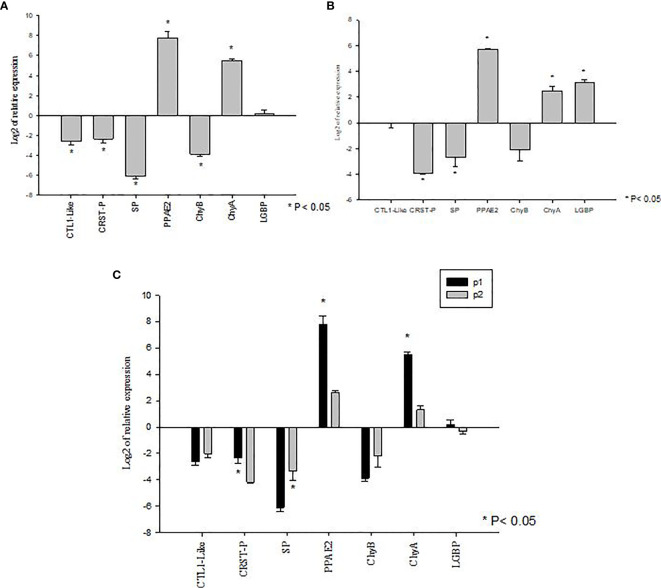
Validation of gene expression profiles of metabolic and immune genes in *Penaeus vannamei* from Bioassay 3 **(A)** The mRNA expression profile in *P. vannamei* AHPND susceptible (P1) population (Panel **A**) and AHPND resistant/tolerant (P2) population (Panel **B**) following experimental challenge. Expression level of each gene is shown relative to the expression in correspond control treatment **(C)** Comparison of gene expression profile from AHPND susceptible (P1) vs AHPND resistant/tolerant (P2) population. Expression levels of each gene are shown relative to the expression in P1 negative control treatment. The data are presented as log_2_ of relative expression ± SD. Statistical significance between control and challenged animals for each of the candidate gene was determined using Student’s t-test. *P<0.05. CRST P, Crustin P; CTL, C-type lectin 1-like; LGBP, Lipopolysaccharide and β-1,3-glucan-binding protein; PPAE2, Prophenol oxidase activation system 2; SP, Serine protease; CHYA, Chymotrypsin A; CHYB, Chymotrypsin B.

The genes showing significant differences in expression levels between susceptible and tolerant shrimp were selected for validations in Bioassay 3. In the P1 population, the expression of CTL1-like, CRSTP, SP and ChyB were significantly down regulated (P<0.05) meanwhile PPAE2 and ChyA expression levels were significantly up regulated (P<0.05) (**Figure 4A**). LGBP expression level was not significant during the experiment (P>0.05) (**Figure 4A**).

In P2 population, PPAE2, ChyA and LGBP expression levels were significantly up regulated (P<0.05). In contrast, the expression levels of CRSTP and SP were down regulated (P<0.05) (**Figure 4B**). CTL1-like and ChyB expression were not significantly different during the experiment (P>0.05) (**Figure 4B**).

By comparison, P1 and P2 challenged animals showed the same expression pattern in both Bioassay 2 and Bioassay 3. The expression levels of CRSTP, PPAE2 and ChyA were significantly higher in P1 population (P<0.05). LGBP expression level was higher in P1 population but not significantly different (P>0.05). Meanwhile, SP had a significant higher expression level in P2 population than P1 population (P<0.05) (**Figure 4C**). ChyB expression level was higher in P2 population than P1 population even though there was no significant difference observed during the experiment (P>0.05) (**Figure 4C**) (**Supplementary Table S3**).

## Discussion

Bacterial pathogenesis in crustaceans is well studied and genes involved in humoral and cellular immunity are known. We decided to take advantage of this background knowledge by measuring the expression of genes that are well known to be involved in defense and metabolic responses during bacterial infections in shrimp. Coincidentally, we had access to AHPND-tolerant lines of *P. vannamei* and considering the lethal nature of AHPND-causing *V. parahaemolyticus* we explored if genes known to be involved in other bacterial pathogenesis in shrimp are also involved in AHPND pathogenesis. To our knowledge, a recently published paper is the first report of the development of AHPND resistant/tolerant lines of *P. vannamei*, Aranguren Caro et al. (15), and as of today, there is no report of looking into the gene expression profiles of AHPND-tolerant *vs*. susceptible lines.

It is now widely accepted that the etiology of AHPND is the insecticidal binary toxin-like genes carried by plasmid DNA in *Vibrio* spp (11, 12, 27). An AHPND resistant/tolerant shrimp line would be ideal in controlling the disease in shrimp aquaculture. Tinwongger and colleagues (16) showed that shrimp exposed to formalin killed cells (FKC) of AHPND causing *V. parahaemolyticus* can survive upon AHPND challenge, and anti-lipopolysaccharide factor AV-R isoform (LvALF AV-R) showed significantly higher expression in the hepatopancreas from the survivor. Interestingly, only four out of two hundred shrimp (2%) survived after feeding with FKC diet, and only three shrimp from the survivor group were used for gene expression analysis. Despite examining a limited number of animals, the authors were successful in identifying genes that could be potentially involved in AHPND pathogenesis. In this study, three bioassays were performed. Bioassay 1 was performed to identify an AHPND tolerant line, P2, using mortality and histopathology as end point data of the bioassay. The P2 population suffered 28% mortality compared to the susceptible line P1 that experienced 87.5% mortality. The bioassay was repeated using the same AHPND-susceptible (P1) and tolerant line (P2) (i.e. Bioassay 2) to examine the mRNA expression of twelve candidate immune and metabolic genes. When the expression of these genes were compared before and after challenge within a population, P1 and P2, a number of genes showed differential expression. However, when the expression profiles were compared between P1 and P2 populations after AHPND-challenge, seven candidate genes showed differential expression. These genes are likely involved in AHPND pathogenesis. In order to further validate the expression of these seven genes, a third bioassay was conducted (i.e. Bioassay 3) and samples were collected for the gene expression validation. The data shed light on the molecular basis of AHPND pathogenesis and enabled to identify potential markers for AHPND tolerance/susceptibility, as discussed below.

### Bioassay 1

The mortality data in the Bioassay 1 showed that the survival rate of the P2 population was over five times higher than P1 population indicating that P2 population is indeed an AHPND tolerant line. The mortality data was consistent with the histopathology findings that cellular damage in the hepatopancreas was far greater in animals from the P1 compared to the P2 population. For example, sloughing of the epithelial cells in the hepatopancreatic tubules followed by massive infiltration of bacterial cells that are considered pathognomonic for AHPND was clearly evident by H&E histology in animals from P1 population (**Figure 1**), whereas in the P2 population, the lesions present in the hepatopancreas resembled more of a chronic infection as seen during SHPN. The differences in mortality and histopathology data led to examining the expression profiles of twelve metabolic and immune-related genes in these two populations. Interestingly, some genes in the tolerant shrimp such as CRST-P, SP, ChyB and LGBP showed the discrepancies in the trend of expression between Bioassay 2 and Bioassay 3. However, only LGBP expression significantly down-regulated in tolerant shrimp from Bioassay 2 meanwhile LGBP expression in tolerant shrimp from Bioassay 3 showed significantly up-regulated. The reasons for discrepancies could be different animals reacting differently to the same pathogen although they are similar genetic line. That would be the reason we had to do validation test with larger number animals.

### Gene Expression in *P. vannamei* From Bioassay 2 *vs*. Bioassay 3

The expression of twelve candidate genes known to be involved in other bacterial diseases were examined to determine if similar genes are involved in AHPND pathogenesis. It was interesting to note that there was no significant difference in the expression levels of *pir*A or *pir*B toxin genes between the P1 and P2 populations suggesting that the animals from the two populations were exposed to equivalent levels of toxin. Thus, the difference in tolerance was most likely due to the difference in immune response between the two populations.

It is known that shrimp, like other invertebrates, elicit cellular and humoral immune responses when exposed to microbes or non-self-protein containing pathogen associated molecular patterns (PAMP) (28, 29). PAMP is easily recognized by pattern recognition proteins including BGBP, LGBP and CTL (19, 30–33). Several studies indicate that the expression of BGBP, LGBP and CTL are up-regulated in shrimp challenged with pathogens such as bacteria, viruses and fungi (34–37).

In Bioassay 2, when the mRNA expressions of BGBP, LGBP and CTL1-like genes were compared between AHPND-susceptible P1 and AHPND-tolerant P2 populations, LGBP and CTL1-like genes were found to be upregulated in P1 compared to P2 population. However, there was no difference in BGBP expression between the two populations (**Figure 3**). Interestingly, in Bioassay 3, although LGBP expression was higher in P1 than P2 populations as in Bioassay 2 samples, the difference in expression was not statistically significant (p=0.365). In contrary, CTL1-like gene did not show any differential expression between the two populations (**Figure 4**), as observed in samples derived from Bioassay 2 (**Figure 3**). These discrepancies highlight two important facts: (i) it is critical to validate gene expression data with biological samples derived from independent bioassays, and (ii) it is important to validate initial findings with larger sample sets, as in Bioassay 3 compared to Bioassay 2. These gives further credence to the mRNA expression findings reported in this study.

In shrimp, upon microbial infection pattern recognition protein(s) circulating in the hemolymph triggers immune response by activating proPO cascade and releasing anti-microbial peptides such as PEN and CRSTP to eliminate the invading pathogen(s) (20). Although PEN and CRSTP were shown to have antimicrobial activities against *Vibrio* spp. and Gram positive bacteria in penaeid shrimp (21, 23, 38), neither of these genes were significantly upregulated in the challenged animals in Bioassay 2. In shrimp, PEN2 is mostly detected in hemocytes and to a lesser level in hepatopancreas. Since we examined the gene expression in hepatopancreas tissue, it is possible that for this reason PEN2 was not found to be differentially expressed. Interestingly, CRSTP which is also predominantly expressed in hemocytes and less in hepatopancreas showed significantly higher expression in AHPND susceptible P1 compared to AHPND tolerant P2 populations in both Bioassays 2 and 3. It remains to be determined if higher CRSTP expression in the susceptible population is due to increased bacterial cell deaths and consequently the release of PirAB^VP^ toxin in the infected animals.

The proPO activation is an important event in crustacean immunity to eliminate pathogens from the circulatory system (39, 40). The final event in the proPO activation process is the conversion of proPO to phenoloxidase (PO) by the PPAE (26, 41, 42). In both Bioassays 2 and 3, PPAE2 showed significantly higher expression after AHPND challenge in P1 compared to P2 populations. The finding is consistent with a previously published report in *P. monodon* where PPAE2 expression in the stomach showed significant upregulation at 24 hour post-challenge with AHPND causing *V. parahaemolyticus* (43). In a separate study, Apitanyasai et al. (44) suggested an overreaction of the proPO cascade causes damage to host cells during AHPND infection and leads to higher mortality. The higher expression of PPAE2 in AHPND susceptible P1 population supports this observation. Taken together, this evidence suggests that upon infection with Vp_AHPND_, not only the expression of antimicrobial peptide like CRSTP but also the genes involved in the proPO pathway are elevated in AHPND susceptible compared to tolerant animals.

It is known that the activation of proPO leads to production of quinones and other intermediate reactions which polymerizes quinones to melanin resulting in pathogen capsulation (45). Quinone, however, also causes cell death by inducing reactive oxygen species (ROS) production (46–48). In crustacean, ROS can be scavenged by an anti-oxidant system involving enzymes of the SOD family (49–51). The EC-SOD expression in the animals studied was not significantly modulated upon AHPND challenge. The proPO cascade is also modulated by a series of protease inhibitors such as KPI and SEP8 to prevent the over activation (52, 53). Again, both KPI and SEP8 mRNA levels in animals from the P1 and P2 populations were not significantly different. It is tempting to speculate that for the lack of modulation of efforts in EC-SOD, proPO cascade activation led to cell toxicity more in P1 compared to P2 population. It is also possible that killing of Vp_AHPND_ cells leads to further release of PirAB toxin from the inactivated bacterial cells exerting lethal effects in genetically susceptible animals.

The involvement of PO activity in the susceptibility of invertebrates due to bacterial toxins has been well documented in many insect species. For example, cabbage loopers (*Trichoplusia ni*) is inherently susceptible to the Cry toxin secreted from *Bacillus thuringiensis*. However, the Cry toxin resistance is negatively correlated with the PO activity in *B. thuringiensis* challenged *T.ni* (54). In addition, the effectiveness of biological insecticide is also higher in lepidopteran species that show high PO activity (55). Recently, it has been reported that during VP_AHPND_ infection resulting in AHPND, reduction in activation of the proPO system by a serine protease inhibitor, *Lv*Serpin7, results in reduction of the toxic effects compared to an unregulated activation of the PO cascade (44).

Apart from the difference in immune gene expression, the expression of metabolic genes also showed differences between healthy and AHPND-challenged animals in each population and between challenged animals in populations P1 *vs* P2. The expression of ChyB and SP were down regulated whereas ChyA expression was up regulated in AHPND challenged shrimp in both P1 and P2 populations and in both Bioassays 2 and 3. These results are consistent with the findings from a recent study in which SP was shown to be upregulated in AHPND tolerant *P. monodon* (18). In addition, Chymotrypsin is upregulated in AHPND susceptible *P. vannamei* after 24 hour of infection (17) which is also in agreement with our findings.

Recently, the crystal structure of PirAB^VP^ toxin shows homology to the structure of the Cry toxin released by *B. thuringiensis* (56), and it is known that the Cry protein is activated by protease enzymes (56–58). The tertiary structure of PirAB^VP^ involves a heterotetrameric interaction between two PirA^VP^ and two PirB^VP^. It has been hypothesized that PirA^VP^ plays a role in receptor binding while PirB^VP^ is involved in pore formation in the cell membrane (59). *In silico* analysis (https://web.expasy.org/peptide_cutter/) showed that the PirA^VP^ toxin contains twelve and nine putative sites that are likely to be cleaved by chymotrypsin and trypsin, respectively (**Supplementary Figure S2**). Meanwhile, PirB^VP^ toxin contains 53 and 36 sites cleaved by chymotrypsin and trypsin, respectively. Multiple alignments between Cry toxin and PirA^VP^ showed that 3 out of 12 and 3 out of 9 cleaved sites for chymotrypsin and trypsin were conserved. Meanwhile, the multiple alignment between Cry toxin and PirB^VP^ showed that 9 out of 53 and 1 out of 36 cleaved sites for chymotrypsin and trypsin were conserved (**Supplementary Figure S2**). It is interesting to note that SP expression showed higher levels in the AHPND tolerant than AHPND susceptible *P. vannamei* in this study and this enzyme belongs to trypsin family (60). It remains to be determined if SP is involved in cleaving PirAB^VP^ toxins to de-activate the toxin. If so, a higher expression of this enzyme in AHPND tolerant population (P2) may prevent the activation of toxin from exerting a lethal effect.

To summarize, we compared the gene expression profiles of two populations of *P. vannamei* that differ in susceptibility to AHPND. The two populations showed a major difference in survival upon experimental challenge. The difference in susceptibility was further evidenced by the differences observed by histopathology. In order to understand the molecular mechanisms governing tolerance and susceptibility, a set of candidate genes that are known to be involved in bacterial pathogenesis and in metabolism in crustaceans were evaluated. Seven genes that showed differential expression in Bioassay 2 were further evaluated in a follow-up challenge, Bioassay 3. The pattern of differential expression between the susceptible (P1) and tolerant (P2) population in Bioassays 2 and 3 were in agreement. Despite the fact that the mRNA expression of only handful genes were measured and a limited number of animals were screened, the information, *albeit* limited, provides valuable insight in *V. parahaemolyticus* pathogenesis and sheds light on how susceptible and tolerant populations of *P. vannamei* respond differently to Vp_AHPND_. To our knowledge, this is the first report looking into the differences in gene expression profiles between AHPND tolerant and susceptible lines in *P. vannamei*.

## Data Availability Statement

The original contributions presented in the study are included in the article/**Supplementary Material**, further inquiries can be directed to the corresponding author.

## Author Contributions

AD conceived the idea. HM and AD designed the study. HM and RC-F performed gene expression analyses. HM wrote the manuscript. LA performed histopathological examine. BN carried out experimental challenge experiment. HM, RC-F, LA, and AD reviewed the manuscript. All authors contributed to the article and approved the submitted version.

## Funding

Funding for this research was provided by Aquaculture Pathology Laboratory Diagnostic Fund. Part of this work is also supported by the USDA National Institute of Food and Agriculture, Hatch/Multistate project 1018120.

## Conflict of Interest

The authors declare that the research was conducted in the absence of any commercial or financial relationships that could be construed as a potential conflict of interest.
