# Supplementary material for: Differentially Expressed Genes in Hepatopancreas of Acute Hepatopancreatic Necrosis Disease Tolerant and Susceptible Shrimp (Penaeus vannamei)
Source: Front Immunol. 2021 May 13;12:634152. doi: 10.3389/fimmu.2021.634152 (PMC8155527; doi:10.3389/fimmu.2021.634152)
Supplement: Supplementary file 1 [file DataSheet_1.docx]

**Supplementary material for differentially expressed genes in hepatopancreas of acute hepatopancreatic necrosis disease (AHPND) tolerant and susceptible shrimp (*Penaeus vannamei*)**

**Hung N Mai^1^, Luis Fernando Aranguren Caro^1^, Roberto Cruz-Flores^1^, Brenda Noble White^1^, and Arun K. Dhar^1*^**

Aquaculture Pathology Laboratory, School of Animal and Comparative Biomedical Sciences, The University of Arizona, 1117 E. Lowell Street, Tucson, AZ, 85721, USA.

*** Correspondence:**Arun K. Dhar
[adhar@email.arizona.edu](mailto:adhar@email.arizona.edu)

# Table S1. The nucleotide sequence of primers used to measure the expression levels of metabolic and immune genes in AHPND-susceptible and -tolerant Penaeus vannamei by quantitative reverse transcriptase-polymerase chain reaction (qRT-PCR).

| Genes | Primers | Sequences (5’ to 3’) | References |
| --- | --- | --- | --- |
| SEP8 | Forward | CCGTGCATATCCGAATTACTCAA | (59) |
|  | Reverse | GCACCATCAGGGCGTTTCTC |  |
| BGBP | Forward | GCTGGCTTACGGCTACGACTT | *This study |
|  | Reverse | GGAGAAGAATGACGAGTCGGTCTA |  |
| CRST- P | Forward | AGCGACTGCAGGTATTGGTG | (60) |
|  | Reverse | TCGTTGGAACAGGTTGTGG |  |
| CTL1-like | Forward | CAAGACCCACAGCGGAGAGA | * This study |
|  | Reverse | TAGACGAGGGCGAAGGTTTC |  |
| KPI | Forward | AGGCAGCTGTTTGCACGAAT | * This study |
|  | Reverse | AGTGCAGTCACAATTGCCATCT |  |
| LGBP | Forward | CATGTCCAACTTCGCTTTCAGA | (34) |
|  | Reverse | ATCACCGCGTGGCATCTT |  |
| EC-SOD | Forward | ATGAAGACGTTGGCAACTCTG | (61) |
|  | Reverse | CTCGCAGGTGGAGTGGAG |  |
| SP | Forward | ACGTTCTCACGACTGGTCACAC | (60) |
|  | Reverse | TATGTAAGGCGCGTCGTTCTC |  |
| PEN2 | Forward | TCGTGGTCTGCCTGGTCTT | (60) |
|  | Reverse | CAGGTCTGAACGGTGGTCTTC |  |
| PPAE2 | Forward | TTCCTTGGGTGGCTGCTTT | (59) |
|  | Reverse | TGTTCGCCGAGACGGATTAC |  |
| ChyA | Forward | AGCCAGCCAGGTCTCCATT | * This study |
|  | Reverse | AAGAGTTCCAGTTCTCGTGAGTGA |  |
| ChyB | Forward | ACCGGCAGTATCTCCAACGT | * This study |
|  | Reverse | TTGCAGTCGTCGTTCGTCAT |  |
| *pirA* | Forward | CAAACGGAGGCGTCACAGA | * This study |
|  | Reverse | GACCGACTTCCGGGATGAT |  |
| *pir*B | Forward | TGCAAACCAAGATAACGTGTATGA | * This study |
|  | Reverse | GCCGTGAACCGTACACCAA |  |
| EF1-α | Forward | TCGCCGAACTGCTGACCAAGA | (62) |
|  | Reverse | CCGGCTTCCAGTTCCTTACC |  |
|  |  |  |  |


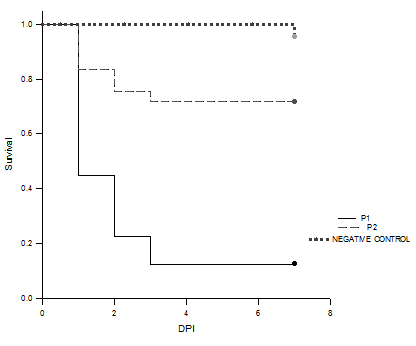


**Figure S1.** Survival rate in two populations of *Penaeus vannamei* from Bioassay 1. P1= Population 1 (Susceptible); P2=Population 2 (Tolerant); SPF=Specific pathogen free; DPI=Days post-infection.

**
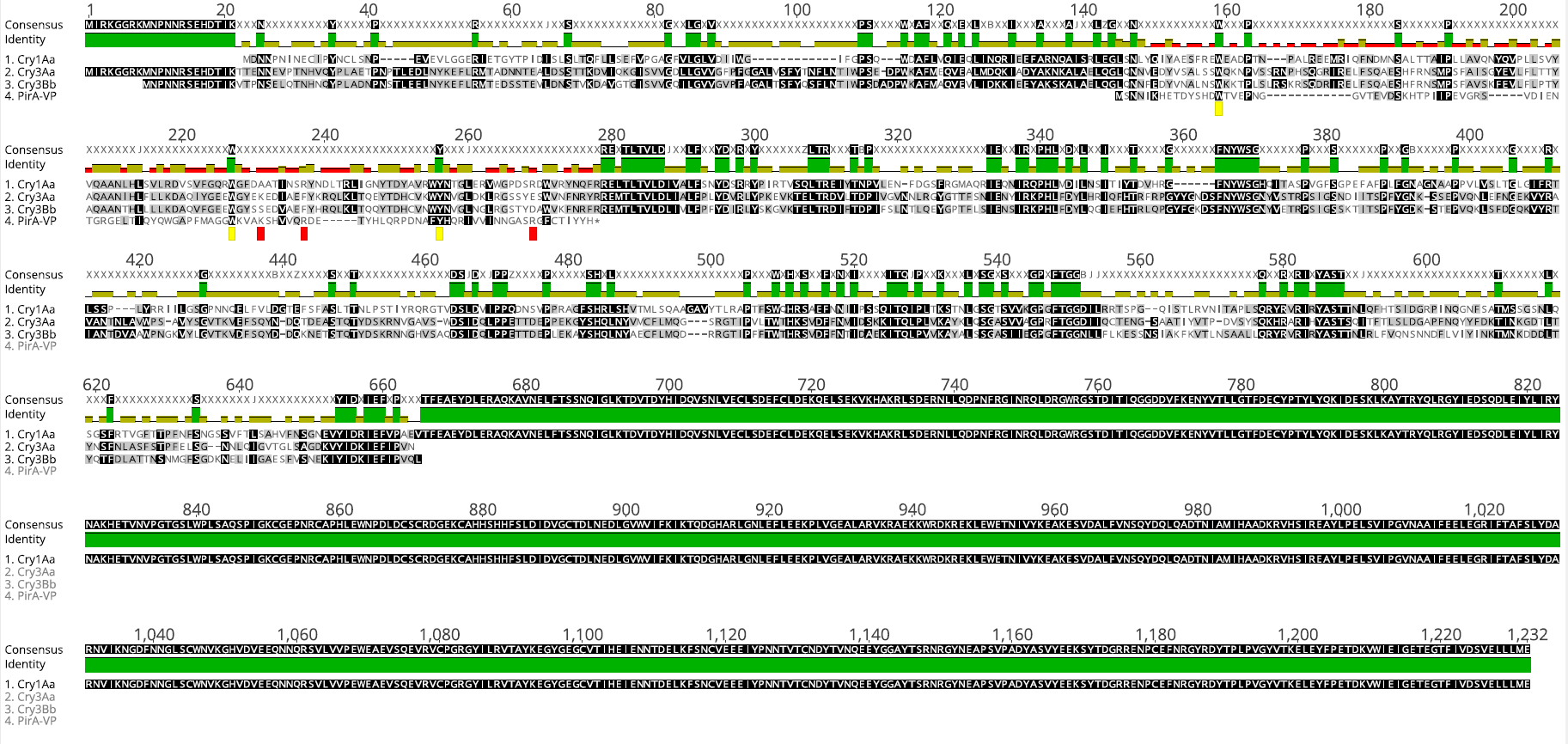

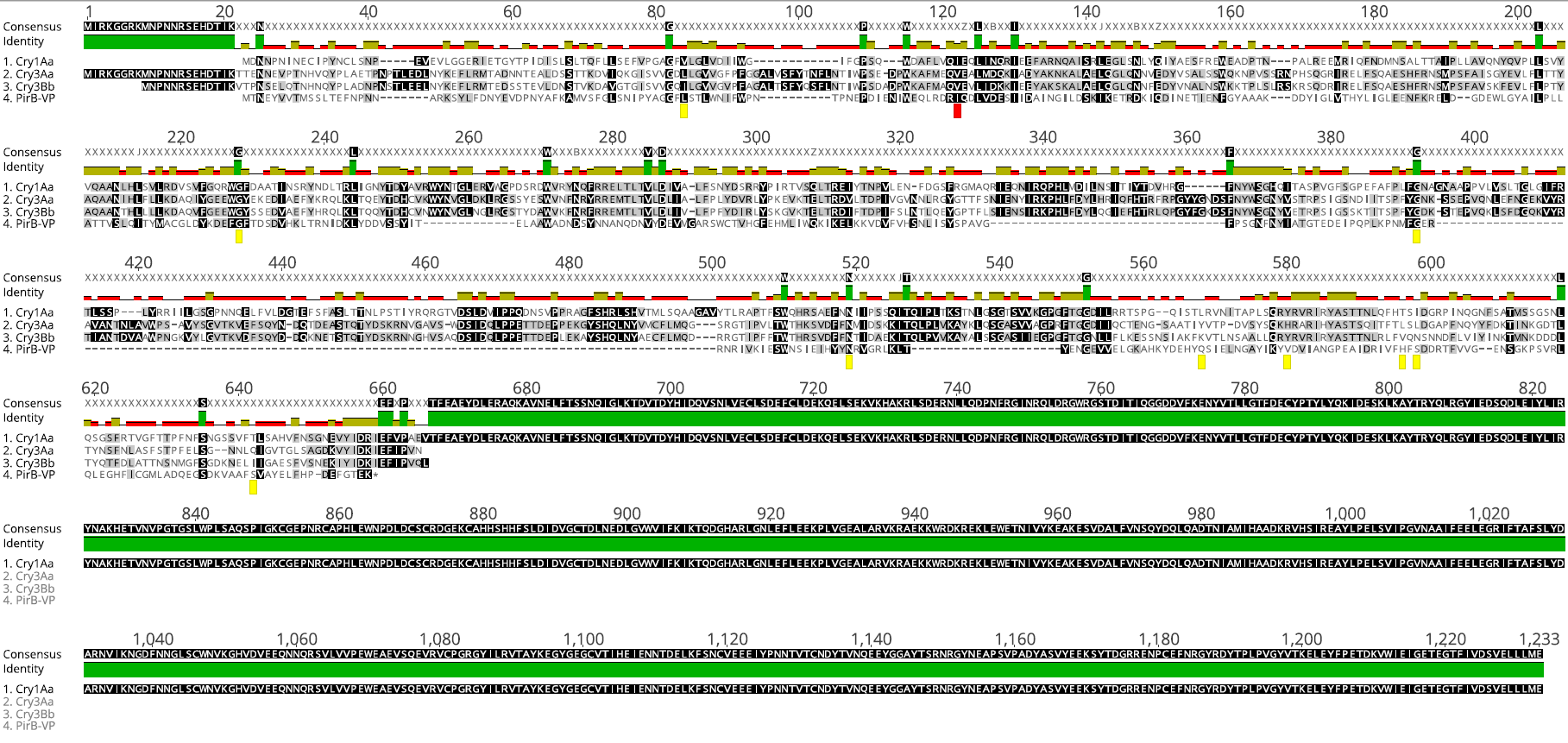
**

A

B

**Figure S2.** Multiple alignment of amino acid sequences of PirA*^VP^* /PirB*^VP^* with Cry1Aa (AEI71570.1), Cry3Aa (AAU29411.1) and Cry3Bb (Q06117.1). (A) Multiple alignment of amino acid sequences of PirA*^VP^* with Cry1Aa, Cry3Aa and Cty3Bb. (B) Multiple alignment of amino acid sequences of PirB*^VP^* with Cry1Aa, Cry3Aa and Cry 3Bb. Green color indicates identical residues. Black color indicates strong similarity. Grey color indicated weak similarity. The cleaved site for chymotrypsin was indicated by yellow. The cleaved site for trypsin was indicated by red color.

Table S2. Differentially expression of 12 immune and metabolic related genes between AHPND susceptible (P1) and AHPND tolerant/resistant (P2) population (2^nd^ bio trial)

ΔCt was calculated as Ct _(target gene)_ – Ct _(EF-1α)_

ΔΔCt was calculated as ΔCt_(challenged animal)_ - ΔCt_(unchallenged animal)_

| Genes | Before challenge-P1 (Avg. ΔCt) | After AHPND Challenge-P1(Avg. ΔCt) | Log_2_2 ^-ΔΔCt^ | P-value |
| --- | --- | --- | --- | --- |
| SEP | 10.23 ± 3.94 | 8.42±1.38 | 1.81±0.60 | 0.418 |
| BGBP | 6.38±1.17 | 6.79±0.77 | -0.41±0.38 | 0.597 |
| ChyA | 2.01±4.12 | -3.19±0.57 | 5.20±0.25 | 0.047^*^ |
| ChyB | 8.23±2.29 | 9.64±1.47 | -1.41±0.63 | 0.340 |
| CRST-P | 4.56±4.29 | 1.68±1.89 | 2.88±0.99 | 0.281 |
| CTL-1-like | 3.79±1.87 | 1.21±1.30 | 2.58±0.77 | 0.093 |
| KPI | 9.04±3.57 | 10.30±8.20 | -1.26±3.80 | 0.804 |
| LGBP | 5.66±0.60 | 3.02±1.22 | 2.64±0.59 | 0.012^*^ |
| PEN2 | 8.80±2.27 | 11.28±6.36 | -2.48±2.78 | 0.494 |
| PPAE2 | 10.30±3.80 | 4.64±1.62 | 5.67±0.70 | 0.034^*^ |
| SOD | 9.96±3.78 | 14.64±5.19 | -4.68±2.25 | 0.827 |
| SP | 10.63±3.79 | 18.95±1.37 | -8.31±0.59 | 0.006^**^ |

| Genes | Before challenge-P2(Avg. ΔCt) | After AHPND Challenge-P2(Avg. ΔCt) | Log_2_2 ^-ΔΔCt^ | P-value |
| --- | --- | --- | --- | --- |
| SEP | 13.86±1.16 | 7.26±3.26 | 6.6±1.41 | 0.057 |
| BGBP | 10.36±5.24 | 9.29±3.19 | 1.07±1.38 | 0.763 |
| ChyA | 5.12±11.2 | 0.43±1.67 | 4.69±0.72 | 0.403 |
| ChyB | 7.25±0.33 | 6.62±0.89 | 0.63±0.39 | 0.278 |
| CRST-P | 11.00±4.29 | 7.56±0.80 | 3.44±0.35 | 0.458 |
| CTL-1-like | 8.52±7.81 | 4.00±1.18 | 4.52±0.51 | 0.563 |
| KPI | 11.74±3.25 | 7.51±2.66 | 4.23±1.15 | 0.158 |
| LGBP | 4.13±0.35 | 9.04±2.48 | -4.91±1.05 | 0.025^*^ |
| PEN2 | 14.17±0.18 | 10.61±4.22 | 3.56±1.83 | 0.324 |
| PPAE2 | 12.41±0.90 | 9.67±2.92 | 2.74±1.26 | 0.286 |
| SOD | 14.17±0.18 | 13.43±2.74 | 0.74±1.18 | 0.739 |
| SP | 14.17±0.18 | 13.43±2.74 | 0.74±1.18 | 0.739 |

| Genes | Before challenge-P1(Avg. ΔCt) | After AHPND Challenge-P1(Avg.ΔCt) | After AHPND Challenge-P2(Avg. ΔCt) | Log_2_2 ^-ΔΔCt^ (P1) | Log_2_2 ^-ΔΔCt^ (P2) | P-value |
| --- | --- | --- | --- | --- | --- | --- |
| SEP | 10.23±3.94 | 8.42±1.38 | 7.26±3.26 | 1.81±0.60 | 2.97±1.41 | 0.540 |
| BGBP | 6.38±1.17 | 6.79±0.77 | 9.29±3.19 | -0.41±0.38 | -2.91±1.38 | 0.184 |
| ChyA | 2.01±4.12 | -3.19±0.57 | 0.43±1.67 | 5.20±0.25 | 1.58±0.72 | 0.006^**^ |
| ChyB | 8.23±2.29 | 9.64±1.47 | 6.62±0.89 | -1.41±0.63 | 1.61±0.39 | 0.012^*^ |
| CRST-P | 4.56±4.29 | 1.68±1.89 | 7.56±0.80 | 2.88±0.99 | -3.00±0.35 | 0.010^*^ |
| CTL-1-like | 3.79±1.87 | 1.21±1.30 | 4.00±1.18 | 2.58±0.77 | -0.21±0.51 | 0.040^*^ |
| KPI | 9.04±3.57 | 10.30±8.20 | 7.51±2.66 | -1.26±3.80 | 1.53±1.15 | 0.581 |
| LGBP | 5.66±0.60 | 3.02±1.22 | 9.04±2.48 | 2.64±0.59 | -3.38±1.05 | 0.009^**^ |
| PEN2 | 8.80±2.27 | 11.28±6.36 | 10.61±4.22 | -2.48±2.78 | -1.81±1.83 | 0.867 |
| PPAE2 | 10.30±3.80 | 4.64±1.62 | 9.67±2.92 | 5.67±0.70 | 0.63±1.26 | 0.024^*^ |
| SOD | 9.96±3.780 | 14.64±5.19 | 13.43±2.74 | -4.68±2.25 | -3.47±1.18 | 0.583 |
| SP | 10.63±3.79 | 18.95±1.37 | 13.43±2.74 | -8.31±0.59 | -2.80±1.18 | 0.011^*^ |

*P<0.05; **P<0.01

Table S3. Differentially expression of 7 immune and metabolic related genes between susceptible (P1) and resistant/tolerant (P2) population (3^rd^ bio trial)

ΔCt was calculated as Ct _(target gene)_ – Ct _(EF-1α)_

ΔΔCt was calculated as ΔCt_(challenged animal)_ - ΔCt_(unchallenged animal)_

| Genes | Before challenge-P1 (Avg. ΔCt) | After AHPND challenge-P1(Avg. ΔCt) | Log_2_2 ^-ΔΔCt^ | P-value |
| --- | --- | --- | --- | --- |
| CTL-1-like | 4.18±0.44 | 6.77±0.52 | -2.59±0.32 | 0.006^**^ |
| CRST-P | -0.17±0.43 | 2.17±0.72 | -2.34±0.44 | 0.017^*^ |
| SP | 2.46±0.47 | 8.55±0.51 | -6.09±0.31 | 0.000^**^ |
| PPAE | 12.79±3.73 | 5.03±1.52 | 7.76±0.64 | 0.047^*^ |
| ChyB | -2.95±0.21 | 0.92±0.41 | -3.87±0.25 | 0.000^**^ |
| ChyA | 8.11±0.18 | 2.63±0.34 | 5.48±0.21 | 0.000^**^ |
| LGBP | 10.21±1.95 | 10.00±0.59 | 0.21±0.36 | 0.889 |

| Genes | Before challenge-P2 (Avg. ΔCt) | After AHPND challenge-P2 (Avg. ΔCt) | Log_2_2 ^-ΔΔCt^ | P-value |
| --- | --- | --- | --- | --- |
| CTL-1-like | 6.15±0.52 | 6.21±0.50 | -0.06 | 0.827 |
| CRST-P | 0.12±0.33 | 4.01±0.17 | -3.89 | 0.045^*^ |
| SP | 3.13±0.29 | 5.80±1.15 | -2.67 | 0.033^*^ |
| PPAE | 15.84±2.38 | 10.14±0.02 | 5.70 | 0.027^*^ |
| ChyB | -2.89±0.28 | -0.79±1.46 | -2.10 | 0.117 |
| ChyA | 9.32±0.90 | 6.82±0.56 | 2.50 | 0.029^*^ |
| LGBP | 13.61±0.74 | 10.51±0.40 | 3.10 | 0.007^**^ |

| Genes | Before challenge-P1 (Avg. ΔCt) | After AHPND challenge-P1(Avg. ΔCt) | After AHPND challenge-P2(Avg. ΔCt) | Log_2_2 ^-ΔΔCt^ (P1) | Log_2_2 ^-ΔΔCt^ (P2) | P-value |
| --- | --- | --- | --- | --- | --- | --- |
| CTL-1-like | 4.18±0.44 | 6.77±0.52 | 6.21±0.50 | -2.59±0.32 | -2.03±0.31 | 0.332 |
| CRST-P | -0.17±0.43 | 2.17±0.72 | 4.01±0.17 | -2.34±0.44 | -4.18±0.10 | 0.025^*^ |
| SP | 2.46±0.47 | 8.55±0.51 | 5.80±1.15 | -6.09±0.31 | -3.34±0.70 | 0.036^*^ |
| PPAE | 12.79±3.73 | 5.03±1.52 | 10.14±0.02 | 7.76±0.64 | 2.65±0.10 | 0.002^**^ |
| ChyB | -2.95±0.21 | 0.92±0.41 | -0.79±1.46 | -3.87±0.25 | -2.16±0.89 | 0.187 |
| ChyA | 8.11±0.18 | 2.63±0.34 | 6.82±0.56 | 5.48±0.21 | 1.29±0.35 | 0.001^**^ |
| LGBP | 10.21±1.95 | 10.00±0.59 | 10.51±0.40 | 0.21±0.36 | -0.3±0.25 | 0.365 |

* P<0.05; **P<0.01

Table S4. A summary of immune and metabolic genes that are known to be expressed early during infection caused by bacteria, virus and fungi in crustaceans.

| Host species | Genes involved | Pathogen | Key findings | References |
| --- | --- | --- | --- | --- |
| *Penaeus vannamei* | Lipopolysaccharide and β-1,3-glucan-binding protein (LGBP) | *Vibiro alginolyticus* | LGBP expression upregulated at 3 hour post injection | ^60^ |
| *Marsupenaeus japonicus* |  | LPS (a pathogen-associated molecule) | LGBP expression upregulated at 12 hour post injection (hpi) | ^61^ |
| *P. vannamei* |  | *V. parahaemolyticus* | LGBP expression upregulated at 12 hpi | ^62^ |
| *P. stylirostris* |  | White spot syndrome virus (WSSV) | LGBP expression start upregulating at 8 hpi | ^63^ |
| *Pacifastacus californiensis* | β-1,3-glucan binding protein (BGBP) | Laminarin (β-1,3-glucan-a pathogen-like) | BGBP activated the pro- phenoloxidase after 30 minutes incubation | ^64^ |
| *P. leniusculus* |  | *Aphanomyces astaci* | BGBP can stimulate the phagocytosis (time course = 6 hours) | ^65^ |
| *P. vannamei* | C-type lectin (CTL) | WSSV | CTL1-like expression increased at 12 hpi | ^66^ |
| *Fenneropenaeus chinensis* |  | *V. anguillarum* and Staphylococcus aureus | Fclectin expression upregulated at 6 hpi | ^67^ |
| *M. japonicus* |  | *V. parahaemolyticus* | CTL expression upregulated at 6 hpi | ^68^ |
| *P. vannamei* | Crustin (CRST) | *V. parahaemolyticus* | Crustin expression up-regulated at 4 hpi | ^69^ |
| *P. vannamei* |  | *V. parahaemolyticus* | Crustin expression up-regulated at 6 hpi | ^70^ |
| *P. vannamei* |  | *V. parahaemolyticus (AHPND)* | Crustin expression up-regulated at 6 hpi | ^71^ |
| *F. chinensis* | Serine protease (SP) | *Vibrio. sp* | SP expression up-regulated at 2 hpi | ^72^ |
| *Procambarus clarkii* |  | *Aeromonas hydrophilla* | SP expression up-regulated at 6 hpi | ^73^ |
| *P. vannamei* |  | *V. anguillarum* | SP expression up-regulated at 12 hpi | ^74^ |
| *M. japonicus* | Serpin (SEP) | *V. anguillarum* | SEP expression up-regulated at 2 hpi | ^75^ |
| *P. vannamei* |  | *V. anguillarum* | SEP expression up-regulated at 6 hpi | ^76^ |
| *P. vannamei* |  | *V. anguillarum* | SEP expression up-regulated at 2 hpi | ^77^ |
| *P. vannamei* | Prophenol oxidase activation system (PPAE) | *V. harveyi* | PPAE expression up-regulated at 2 hpi | ^78^ |
| *P. vannamei* |  | *V. anguillarum* | PPAE expression up-regulated at 12 hpi | ^74^ |
| *Scylla paramamosain* | Chymotrypsin | *V. alginolyticus* | Chymotrypsin expression up-regulated at 3hpi | ^79^ |
| *Procambarus clarkii* | Kazal type serine protease inhibitor (KPI) | *V. anguillarum* | KPI expression up regulated at 12 hpi | ^80^ |
| *P. monodon* |  | WSSV | KPI expression up regulated at 3 hpi | ^81^ |
| *P. vannamei* | Extracellular Copper/Zinc Superoxide dismutase (EC-SOD) | *V. alginolyticus* | EC-SOD expression up regulated at 3 hpi | ^82^ |
| *M. japonicus* |  | *V. penaeicida* | EC-SOD expression up regulated at 3 hpi | ^83^ |
| *Fenneropenaeus merguiensis* | Penaidin (PEN) | *V. parahaemolyticus* | PEN expression up regulated at 4 hpi | ^84^ |
| *F. indicus* |  | *V. parahaemolyticus* | PEN expression up regulated at 6 hpi | ^85^ |
